# A Comparative Approach Linking Molecular Dynamics of Altered Peptide Ligands and MHC with *In Vivo* Immune Responses

**DOI:** 10.1371/journal.pone.0011653

**Published:** 2010-07-19

**Authors:** Bernhard Knapp, Ulrich Omasits, Wolfgang Schreiner, Michelle M. Epstein

**Affiliations:** 1 Department for Biomedical Computer Simulation and Bioinformatics, Medical University of Vienna, Vienna, Austria; 2 Division of Immunology, Allergy, and Infectious Diseases, Department of Dermatology, Medical University of Vienna, Vienna, Austria; Science Commons, United States of America

## Abstract

**Background:**

The recognition of peptide in the context of MHC by T lymphocytes is a critical step in the initiation of an adaptive immune response. However, the molecular nature of the interaction between peptide and MHC and how it influences T cell responsiveness is not fully understood.

**Results:**

We analyzed the immunological consequences of the interaction of MHC class II (I-A^u^) restricted 11-mer peptides of myelin basic protein with amino acid substitutions at position 4. These mutant peptides differ in MHC binding affinity, CD4^+^ T cell priming, and alter the severity of peptide-induced experimental allergic encephalomyelitis. Using molecular dynamics, a computational method of quantifying intrinsic movements of proteins at high resolution, we investigated conformational changes in MHC upon peptide binding. We found that irrespective of peptide binding affinity, MHC deformation appears to influence costimulation, which then leads to effective T cell priming and disease induction. Although this study compares *in vivo* and molecular dynamics results for three altered peptide ligands, further investigation with similar complexes is essential to determine whether spatial rearrangement of peptide-MHC and costimulatory complexes is an additional level of T cell regulation.

## Introduction

A productive interaction between the peptide antigen (Ag) : MHC class II complex on the surface of an Ag presenting cell (APC) and the T cell receptor (TCR) on a CD4^+^ T lymphocyte leads to an immune response. However, the ability of peptide : MHC class II (pMHC) to activate T cells depends upon many factors including the stability of the complex, TCR : pMHC interaction kinetics, the density of interacting TCRs, amongst other factors [1]. In certain instances, altered peptide ligands (APLs) derived by substituting key amino acids lead to dissociation of effector T cell functions such as proliferation, cytokine production, and disease induction or cause immune tolerance [2], [3] and might have potential therapeutic value for immune-mediated diseases. An example of such an APL is the alanine substitution at position 4 of the I-A^u^ restricted N-terminal 11-mer peptide of myelin basic protein (MPB) (AcN1-11[4A]), which is a poor immunogen that inhibits the induction of the MPB-specific CD4^+^ T cell-mediated experimental allergic encephalomyelitis (EAE) model of multiple sclerosis in mice [4]. The native N-terminal MBP peptide with a lysine at position 4, AcN1-11 is characterized by a low binding activity [5], stimulates MBP-specific T cell clones, primes for *in vivo* recall proliferative responses, cytokine production, and induces EAE in H-2^u^ mice [6], [7]. However, a single amino acid substitution at position 4 changes the binding affinity of the peptide to I-A^u^ and alters MBP-specific T cell responsiveness [8], [9]. The binding affinity of AcN1-9[4A] 9-mer to I-A^u^ (IC50  = 0.019 mM) is higher than AcN1-9 (IC50  = 7.4 mM), it stimulates MBP-specific T cell clones better than the native peptide, but does not induce EAE and diminishes the severity of EAE induced by the native peptide [4], [8]. In contrast, the methionine substitution, AcN1-9[4M], binds I-A^u^ (IC50  = 0.00064 mM) more avidly than AcN1-9 and is a good immunogen [4], [8], [9], illustrating that binding affinity with MHC class II may not correlate with immune responsiveness and suggests that additional mechanisms may be involved.

The intrinsic motions of proteins, determined by covalent and non-covalent forces, cause conformational changes with intermolecular and intercellular ramifications on signaling pathways, cell function, and physiological responses. Molecular dynamics (MD) is a computational approach used to examine conformational dynamics of molecules at high resolution in space and time [10]. Because the binding affinities of APLs to MHC do not accurately predict *in vivo* immunogenicity, we sought to evaluate peptide and MHC interaction dynamics and correlate these movements and conformational changes with functional immunological consequences. Since it has already been shown that conformational differences between peptide : MHC complexes can explain the binding characteristics of MHC class I ligands [11], alloreactive phenomena [12] or the recognition of MHC class II binding epitopes [13], we suggest that the spatial dynamics of MD can further reveal aspects of T cell activation.

## Materials and Methods

### Mice

Female (PL/J × SJL) F1 mice (6–8 weeks old) were purchased from Jackson laboratories (Bar Harbor, ME) or were bred at the Yale University animal facility (New Haven, CT). The animal experiments were conducted at Yale University and passed the “Yale University Institutional Animal Care and Use Committee” (IACUC). All animal studies were performed in accordance with the guidelines of the IACUC.

### Antibodies

The mAbs were purified from the hybridoma supernatants maintained in the lab. The following mAbs used in this study: GK 1.5 (rat IgG2b, anti-CD4), TIB105 (rat IgG2a, anti-CD8 clone 53–6.72), Y3JP (mouse IgG2, anti-I-A^u^), Y19 (anti-Thy 1), 14.4.4s (mouse IgG2a, anti-I-E), 37.51 (hamster IgG, anti-CD28) obtained from Pharmingen (San Diego, CA) or 37.51 ascites kindly provided by J. Allison (Sloan-Kettering, NY).

### Preparation of MBP Peptides

Peptides were synthesized using a solid-phase peptide synthesizer (430A; Applied Biosytems, Inc., Foster City, CA), purified and characterized by high pressure liquid chromatography (HPLC) and mass spectrophotometry. Each peptide ran as a single peak on HPLC and had the predicted amino acid content and molecular weight by mass spectrophotometry. Peptide sequences are: AcN1-11:ASQKRPSQRHG; AcN1-11[4A]:ASQARPSQRHG; AcN1-11[4M]:ASQMRPSQRHG. The Ac denotes N-terminal acetylation.

### T cell clones and lines

The CD4^+^ T clone 19 kindly provided by C. A. Janeway (Yale University) was maintained by stimulation with 6×10^6^ irradiated (2700 rad) syngeneic spleen cells per T-75 flask, plus AcN1-11 (5 µg/ml), and 5 U/ml recombinant murine IL-2 (rIL-2) (Boehringer Mannheim Corporation, Indianapolis, IN).

### MBP peptide induced EAE

We injected each peptide (200 µg) dissolved in PBS and emulsified in Complete Freund's Adjuvant (CFA) containing 4 mg/ml *Mycobacterium tuberculosis* H37RA (Difco Laboratories Inc., Detroit, MI) subcutaneously in the flanks (100 µg each flank) on day 0 followed by two intravenous doses of 200 ng *Bordetella pertussis* toxin given on days 0 and 2 (List Biologicals, CA). We observed animals daily and graded for clinical disease in a blinded fashion. Mean maximum severity scores for EAE were done using the following grading criteria: Grade 1. limp tail; grade 2. limp tail and weak hind limbs; Grade 3. hind limb paralysis; Grade 4. fore limb paralysis; Grade 5. moribund; Grade 6. death. To test the effect of anti-CD28 on disease induction, we injected mAb (100 µg/100 µl) intraperitoneal (i.p.) on days 1, 3, 5, 7 and 9.

### Proliferation assays

MBP peptides (200 µg) were dissolved in PBS and emulsified with an equal volume of CFA containing 4 mg/ml *Mycobacterium tuberculosis* H37RA. For testing the effect of *in vivo* anti-CD28, we injected purified anti-CD28 (100 µg/100 µl) i.p. at the time of immunization and at day 3, 5 and 7. Ten days after the animals were immunized subcutaneously (50 µg/site) at the tail base, draining inguinal and paraaortic lymph nodes were removed. We purified CD4^+^ T cells using immunomagnetic negative selection in which the lymph node cells were incubated with anti-CD8 (TIB 105), anti-H-2^u^ (Y3JP) antibodies in Hanks buffered salt solution (HBSS) at 4°C for 1 h, followed by incubation with goat anti-mouse and goat anti-rat IgG magnetic beads (Collaborative Research Incorporated, Bedford, MA) at 4°C for 1 h. Magnetic selection yielded 95–97% pure CD4^+^ T cells analyzed by flow cytometry. APCs were prepared by incubating splenocytes from non-immunized mice with anti-CD8 (TIB 105), anti-CD4 (GK 1.5) and anti-Thy 1 (Y19) for 1 h at 4°C, following the removal of erythrocytes by density gradient centrifugation with lymphocyte separation media. We then incubated antibody coated cells with rabbit complement at 37°C for 30 min causing complement-mediated killing, yielding 92–95% pure MHC class II bearing cells demonstrated by FACS analysis.

For proliferation assays, mitomycin C-treated APCs (2×10^5^) per well and CD4^+^ T cells (2×10^5^) or clone 19 cells (1×10^5^) in a total 100 µl of Eagles high amino acid media (EHAA) supplemented with 5% FCS, 50 U/ml penicillin, 50 µg/ml streptomycin sulfate, and 10 mM Hepes were plated in triplicate on a 96-flat bottom well microtitre plate (Costar, Cambridge, MA) with peptide or media alone for 5 days at 37°C, 5% CO2, and humidified air. rIL-2 or anti-CD28 ascites or isotype-matched control ascites were added to secondary cultures at the time of the initial setup to maintain a total of 100 µl/well for *in vitro* assay. We added 1 µCi [^3^H]-thymidine (6.7 Ci/mmol, ICN Biomedicals Inc., Irvine, CA) per well at 96 h and harvested 18–24 h later. Results are presented as averages of triplicates and standard errors were <20% unless otherwise indicated.

### Cytokine Assays

Bioassays for IFNγ were done by standard protocols, in which we incubated 40 µl of culture supernatant with 20 µl (5000 cells/well) of WEHI-279 indicator cells (ATCC, Rockville, MD) in the presence and absence of anti-IFNγ to measure IFNγ and pulsed cultures with [^3^H]-thymidine at 24 h and harvested 24 h after. One unit of IFNγ is the concentration, which gave half maximal inhibition of WEHI-279 proliferation calculated from the standard curve obtained with a murine rIFNγ standard (Genzyme Corp., Boston, MA). In all assays, anti-IFNγ blocked inhibition.

### Molecular dynamics

MD studies started with the crystallographic pMHC structure (pdb-id 1k2d [14]), containing a peptide which is most similar to our target MBP AcN1-11 : I-A^u^ (compared to pdb-id 2p24 and pdb-id 1u3h containing less related peptides and pdb-id 2pxy containing TCR fragments making all these less optimal choices). Therefore, using pdb-id 1k2d, we could model MBP AcN1-11, AcN1-11[4A], and AcN1-11[4M] peptides into the binding groove of the MHC molecule in only 4 steps, 1.) removed the four N-terminal residues whose backbones are unnecessary for the modeling process, 2.) attached the missing three C-terminal residues using SPDBV [15], 3.) modeled an acetyl-group N-terminally to the peptide, and 4.) mutated the tyrosine at position 4 to lysine, alanine, and methionine using the SCWRL software [16]. Subsequently, MD simulations are carried out using Gromacs [17]; We immersed each modeled structure into a 3D water cube of 127 Å side length allowing for a minimum distance of 20 Å between protein and box-boundary [18] and applied periodic boundary conditions. Subsequently, we minimized each system energetically using a steepest descent method and then warmed up the systems to 310 K. MD simulations are carried out for a simulation time corresponding to approximately 22 ns of real time for each system. We used Gromacs to analyse the simulations and resulting trajectories [17]. We used built-in analyses tools of Gromacs for root mean square deviation (RMSD) evaluations.

## Results

### AcN1-11 induces dramatic changes in the α-helical conformation of residues 65–69 of the MHC α_1_-chain (r65-69α)

**Figure 1:**
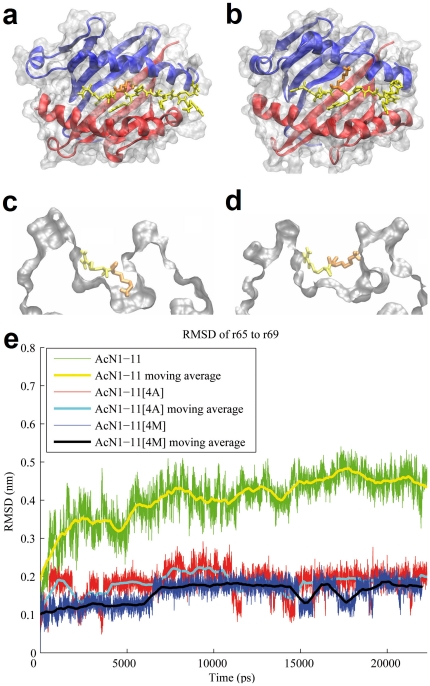
Deformation of r65-69α of the MHC. (a) Top view onto the modeled MBP AcN1-11 : I-A^u^ complex. The MHC α-chain (blue), and the MHC β-chain (red) are represented as a cartoon. The peptide (yellow), represented in licorice style, is bound to the MHC at the peptide binding groove. The lysine at position 4 (orange) is buried in MHC pocket 6. (b) Average structure during simulation from time t = 15 ns to t = 22 ns. The lysine at position 4 left the groove and is bound to the lateral helix of the MHC α-chain, causing a disruption of the helical structure next to position 4 and a widening of the groove at the N-terminal half (i.e. the left side of the picture). (c) Thin cross-section through the MHC pocket 6 of the modeled complex. (d) Same as C but for the average structure during simulation from time t = 15 ns to t = 22 ns. (e) Moving average RMSD of residues 65–69 of the α_1_-helix of the MHC. Ac1-11 causes the largest deformation in this area of the α_1_-helix compared to Ac1-11[4A] and Ac1-11[4M]. The moving average is calculated using the neighboring 1000 frames (500 on the left and 500 on the right).

To evaluate the effects of peptide binding on MHC class II structure, we used the *in silico* MD method to calculate spatial changes of the side-chain of the native AcN1-11 peptide in MHC pocket 6 (Fig. 1a and 1c). The most similar experimental I-A^u^ crystal structure available contains the AcN1-11[4Y] peptide with a tyrosine occupying position 4 [14]. Upon *in silico* substitution of native peptide for AcN1-11[4Y] at the start of MD, we observed that within the first nanosecond (ns) of simulation, AcN1-11 leaves MHC pocket 6 and interacts with the lateral α-helix of the MHC α-chain (Fig. 1b and 1d). Thereby, inducing a distortion of a span of 5 residues (65-69α) in the α -helix of the MHC, causing a widening of the peptide binding groove without affecting the overall structure of the peptide backbone (Fig. 1b and 1d).

To quantify the deformation of the 65–69 amino acid span of the MHC α_1_-helix, which directly neighbors position 4 of the peptide, we calculated the running average RMSD of this region, separately for each peptide (Fig. 1e). To focus on (internal deformations of) the α_1_-helix conformation, excluding the overall movement of the structure, we fitted the simulation trajectory to the neighboring 10 residues (5 on each side) in the α_1_-helix and calculated RMSDs. These data confirm that AcN1-11 causes the largest deformation of the r65-69α span of the α-helical structure compared to the other peptides (Fig. 1e). This is also illustrated in the rendered images Fig. 1b and 1d, where AcN1-11 disrupts the α_1_-helix at G67, which is the center of deformation of the MHC and affects the neighboring A65, T66, K68, and H69 residues as well. We used Visual Molecular Dynamics (VMD, [19]) to illustrate the behavior of lysine at position 4 in a sequence of rendered images during the 22 ns of movement of the peptide within MHC pocket 6 (Movie S1). The average RMSD value over the entire simulation period was 0.404 nm for AcN1-11, whereas AcN1-11[4A] and AcN1-11[4M] averaged only 0.185 nm, and 0.156 nm, respectively. These data demonstrate that AcN1-11 induces a spatial rearrangement in the MHC α_1_-helix that alters the r65-69α span so that it does not conform with the definition of an α-helical structure [20] (Fig. 1b).

### AcN1-11 and AcN1-11[4M] but not AcN1-11[4A] deform the MHC

**Figure 2:**
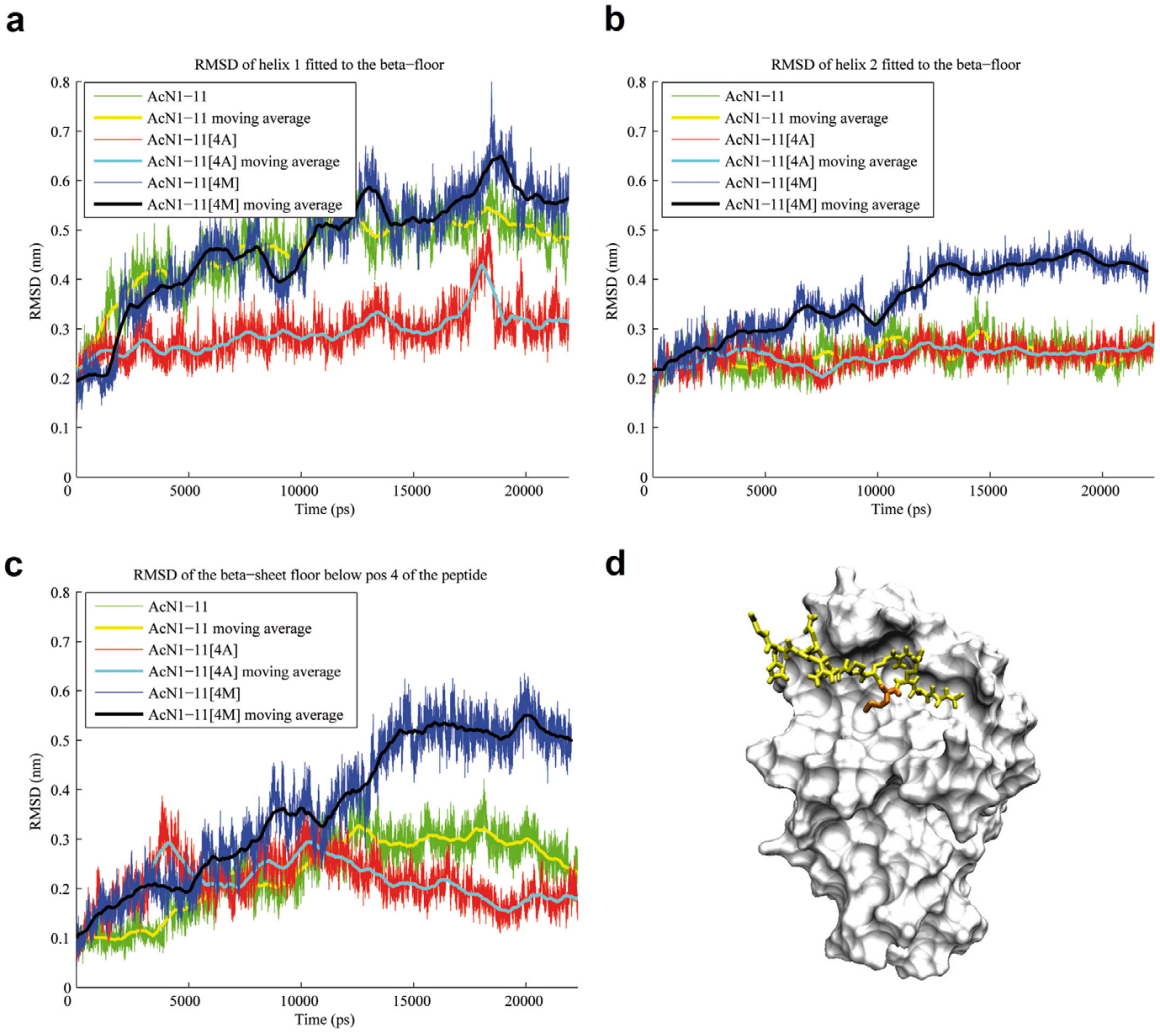
Structural rearrangements of the MHC. (a) Deformation of the α_1_-helix of the MHC. The average RMSD of AcN1-11 and AcN1-11[4M] are similar although the RMSD for AcN1-11 primarily results form deformation of residues 65 to 69, while AcN1-11[4M] deforms the whole helix. (b) Deformation of the α_2_-helix of the MHC. Only Ac1-11N[4M] affects the structure of the α_2_-helix. (c) Deformation of the ß-sheet floor below position 4 of the peptide. AcN1-11[4M] deforms the floor at a much higher level than the other two derivates. (d) 3D representation of the complex. The methionine side-chain stays in pocket 6 over the whole simulation time. The α1-helix is hidden to improve visualization of the binding groove. White: MHC. Yellow: peptide. Orange: Ac1-11[4M].

RMSD evaluations pertaining to the entire α_1_- and α_2_- helices show a different picture. AcN1-11[4M] deformed the overall spatial arrangement of the α_1_- and α_2_- helices and AcN1-11[4A] caused almost no deformation (Fig. 2A). The average RMSD value for AcN1-11[4A] is 0.295 nm, whereas AcN1-11 and AcN1-11[4M] are similar at 0.459 nm and 0.469 nm, respectively. Interestingly, the RMSDs for AcN1-11 and AcN1-11[4M] are similar despite the fact that AcN1-11 only deforms a small span (r65-69α), whereas AcN1-11[4M] causes overall deformation of the helices by influencing almost all of the residues of the MHC α_1_-helix. These data show that AcN1-11[4M] and AcN1-11 induce structural changes to the entire MHC class II molecule not observed with AcN1-11[4A]. This picture is accentuated when evaluating the entire MHC structure, including the β-chain. We observed that AcN1-11[4M] not only induced changes in the α_1_-helix but also affected the α_2_-helix of the MHC β-chain (Fig. 2b), leading to a higher average RMSD of 0.362 nm compared to AcN1-11 and AcN1-11[4A], which average 0.248 nm and 0.246 nm, respectively. Additionally, AcN1-11[4M] is the only peptide capable of deforming the β-sheet floor below position 4 of the peptide (4^th^ strand of the β-sheet, r185–190 of the β-chain) (Fig. 2c), with average RMSDs for AcN1-11[4M] of 0.367 nm, AcN1-11 of 0.233 nm, and AcN1-11[4A] of 0.211 nm. These data show that both AcN1-11[4M] and AcN1-11 differ from AcN1-11[4A] by deforming the α_1_-helix, and that AcN1-11[4M] differs from AcN1-11[4A] and AcN1-11 by deforming both the α_2_-helix and β-sheet.

### Structural rearrangements and experimental binding affinities

Unlike the binding of AcN1-11 to MHC, where the side-chain of lysine at position 4 immediately leaves MHC pocket 6, the side-chains at position 4 of AcN1-11[4A] and AcN1-11[4M] stay inside MHC pocket 6 for the whole simulation time. The side-chain of AcN1-11[4M] wedges into pocket 6 forming an anchor (Fig. 2d), whereas the side-chain of AcN1-11[4A] is too small to be an anchor. These data predict that AcN1-11[4A] and AcN1-11[4M] would have higher binding affinity than the native peptide to MHC and that the anchoring of AcN1-11[4M] would further increase binding affinity, which agrees with measured binding affinities. Despite the lack of position 4 side-chain anchoring of AcN1-11[4A] and avid binding, there is almost no conformational change induced in the MHC, which results in increased MHC stability.

### AcN1-11 and AcN1-11[4M] but not AcN1-11[4A] induce EAE

To compare MD simulations with *in vivo* immunogenicity, we primed H-2^u^ mice with each MBP peptide and adjuvant and observed mice for disease one week after immunization. Table 1 shows that AcN-1-11[4A] does not induce disease, but the native peptide and the AcN-1-11[4M] peptide could generate EAE. Moreover, AcN-1-11[4M] induced more severe disease in 100% of mice compared to disease induction in 80% of mice immunized with the native peptide.

**Table 1:** Severity of EAE upon immunization with MPB AcN1-11 substituted peptides*.

Peptide	Incidence (%)	Mean onset (days)	Mean maximum severity score
AcN1-11[4A]	0	0	0
AcN1-11	80	13	3.4
AcN1-11[4M]	100	14	4.0

*n = 10 mice/group, 4 experiments.

### AcN1-11 and AcN1-11[4M] prime for proliferation and cytokines but AcN1-11[4A] primes only for IFNγ production

**Figure 3:**
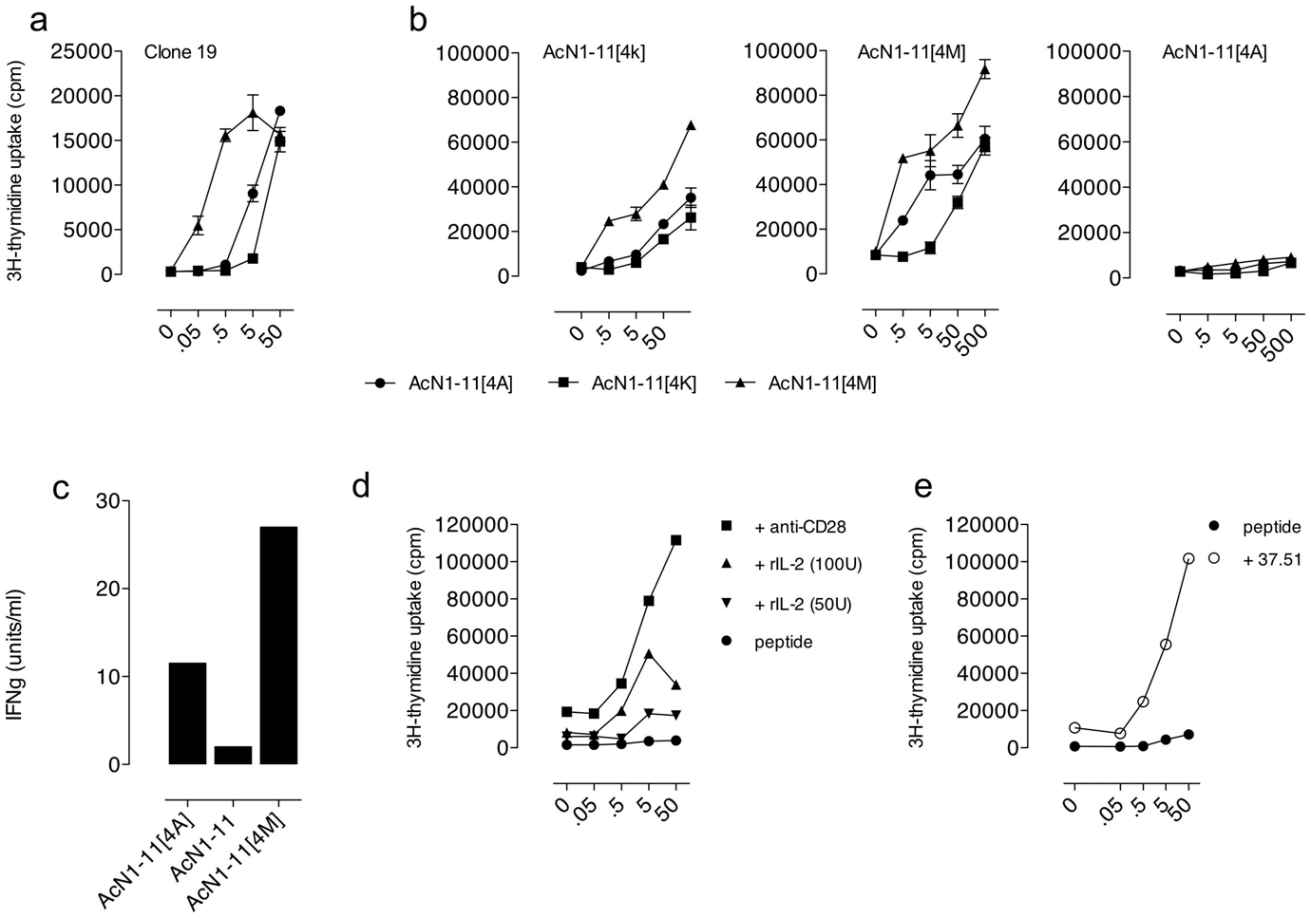
MBP-specific CD4^+^ T cell responses to native and altered peptide ligands. (a) MBP-specific clone 19 cells were incubated with splenic APCs and peptides or media alone for 96 h. Proliferation was measured 18 h after ^3^[H]-thymidine was added to cultures. (b) CD4^+^ T cells from mice immunized with either AcN1-11, AcN1-11[4A] or AcN1-11[4M] were incubated with peptides and splenic APCs. Proliferation was measured 18 h after ^3^[H]-thymidine was added to cultures. (c) Supernatants (96-h) from AcN1-11, AcN1-11[4A], and AcN1-11[4M]-primed CD4^+^ T cells cultured with AcN1-11, AcN1-11[4A], AcN1-11[4M] or media alone, and splenic APCs were tested for IFNγ by bioassay. (d) AcN1-11[4A] primed CD4^+^ T cells were cultured for 96 h in the presence of 100 or 50 Units/ml rIL-2, anti-CD28 ascites (1:2000 dilution) or media and splenic APCs. Proliferation was measured 18 h after ^3^[H]-thymidine was added to cultures. (e) CD4^+^ T cells from mice immunized with AcN1-11[4A] followed by i.p. anti-CD28 or PBS were incubated with splenic APCs, AcN1-11[4A] or media alone. Proliferation was measured 18 h after ^3^[H]-thymidine was added to cultures. These data are mean ± SEM. n = 5 mice per group, with a minimum of 3 experimental repetitions.

To further characterize *in vivo* peptide responses, we immunized mice with individual peptides and cultured purified CD4^+^ T cells from draining lymph nodes with all peptides in the presence of APCs. All three peptides are capable of re-stimulating MBP-specific CD4^+^ T cell line, clone 19 with AcN1-11[4A] and AcN1-11[4M] inducing higher proliferative responses than the native peptide (Fig. 3a). However, when the peptides are used to stimulate *in vitro* secondary responses from peptide-primed CD4^+^ T cells, primed AcN1-11[4A] cells are unable to mount a proliferative recall response to any of the peptides suggesting that *in vivo* priming did not occur (Fig. 3b). In contrast, AcN1-11 and AcN1-11[4M] primed CD4^+^ T cells, like Clone 19, respond to AcN1-11[4M] better than AcN1-11 and AcN1-11[4A]. To rule out that the kinetics of priming and dose of peptide were not responsible for the lack of *in vivo* priming by AcN1-11[4A], we tested AcN1-11[4A] primed CD4^+^ T cells after repeated immunizations and a range of doses from 0.0005 µg to 500 µg and found no response in recall to MBP peptides despite a normal proliferative response to mitogens, concanavalin A, and purified protein derivative (data not shown). Although AcN1-11[4A] immunized mice generated CD4^+^ T cells unable to proliferate upon secondary Ag rechallenge, it was possible that the cells generated were effector cells able to produce cytokines. To test this possibility, we stimulated each peptide-immunized CD4^+^ T cell population with a secondary *in vitro* challenge of the immunizing peptide and found that AcN1-11[4A]-primed CD4^+^ T cells produced IFNγ and that the amount secreted followed the binding affinity with IFNγ levels; AcN1-11[4M] > AcN1-11[4A] > AcN1-11 (Fig. 3c). No CD4^+^ T cells produced IFNγ when cell populations were incubated with medium alone (data not shown). Taken together, these data illustrate the peculiar behavior of AcN1-11[4A] primed CD4^+^ T cells. AcN1-11[4A] neither primes for secondary *in vitro* proliferation or *in vivo* disease, but primes for Ag-specific IFNγ production. This suggests that the alanine substituted peptide causes partial CD4^+^ T cell priming.

### Anti-CD28 restores AcN1-11[4A] proliferation

Because AcN1-11[4A] could prime for cytokine production but not for T cell proliferation, we hypothesized that priming for proliferation and cytokine production vs. cytokine production alone may be related to insufficient costimulatory signals (via CD28) and/or growth factors, such as IL-2, which is a growth factor crucial for T cell proliferation. We tested this hypothesis by adding either anti-CD28 or rIL-2 to *in vitro* cultures to test whether we could reconstitute effective T cell priming. We added titrated doses of rIL-2 or anti-CD28 *in vitro* to AcN1-11[4A] primed CD4^+^ T cells and recall peptide. We found that both rIL-2 and anti-CD28 restored proliferation if the mice were primed with AcN1-11[4A] (Fig. 3d). Mice immunized with PBS/CFA alone (without peptide) were unable to proliferate in the presence of peptide, rIL-2 or anti-CD28 (data not shown).

Since costimulation reconstituted proliferation of partially primed AcN1-11[4A]-specific CD4^+^ T cells *in vitro*, we tested the effect of the addition of costimulation upon initial *in vivo* priming. We observed that i.p. injections with anti-CD28 (clone 37.51) following priming with AcN1-11[4A] proliferated to AcN1-11[4A] *in vitro* compared to the PBS injected control (Fig. 3e). Thus, not only could anti-CD28 and the addition of rIL-2 *in vitro* reconstitute proliferation, but it was also possible to restore recall proliferation during the priming event. We then proceeded to test the possibility that the addition of anti-CD28 *in vivo* during disease induction would induce EAE. Several doses of anti-CD28 were injected following AcN1-11[4A] priming, but EAE could not be generated (data not shown). While AcN1-11[4A] CD4^+^ T cells are able to proliferate in response to AcN1-11[4A] *ex-vivo*, additional factors may be necessary for these cells to track to the central nervous system (CNS) and induce disease and the dose of costimulation may not be high enough to effectively induce pathogenic lymphocytes.

## Discussion

Here, we present an approach to elucidate potential mechanisms underlying molecular interactions that explain crucial immunological phenomena by combining experimental and computational data. MD have shed insights on the intrinsic dynamic properties of proteins by illustrating that binding events influence molecular, cellular, and intercellular interactions and that these effects direct signaling networks [21]. It allows measurements of protein movements in angstrom distances providing new information about intermolecular interactions, which potentially relate to *in vivo* functional outcomes.

For the APLs presented in this study, our data compare dynamics of three peptides within the MHC groove and the consequent conformational changes of MHC, which appears to influence costimulation, T cell activation, and EAE induction. These and other MD simulations provide a potentially new mechanism underlying why peptide affinity to MHC alone does not sufficiently explain the exceptional behavior of APLs like AcN1-11[4A]. Our data suggest that *in vivo* effects correlate with spatial rearrangements between peptide and MHC. These data illustrate on a subset of well-studied peptides that MD simulations could provide interesting insights into the relationship between spatial dynamics of pMHC interactions and immunological outcomes.

Further investigations using MD simulations will be critical for determining whether this approach occurs only with certain classes of peptide or is a broader phenomenon. It is necessary to study a whole range of pMHC interactions, comparing MD simulations with *in vivo* data to determine the generalizability of molecular mechanisms through which conformational changes in MHC molecules are translated into alterations in T cell proliferation and function. Nevertheless, well-tailored MD simulations that correlate with functional immunological outcomes [22], [23], [24], [25], [26], [27], [28], [29] may be useful for designing therapeutic APLs to treat diseases such as multiple sclerosis [30], [31], [32].

## Supporting Information

Movie S1 Movie illustrating the loss of helical structure. The side-chain of AcN1-11 (orange) of the peptide backbone (yellow) disrupts the alpha -helix at r65-69alpha span (green) of the helical structure of the alpha-chain of MHC (blue). beta-chain is depicted in red.
(3.70 MB MOV)
